# Revisiting regulatory T cells for stroke therapy

**DOI:** 10.1172/JCI161703

**Published:** 2022-08-01

**Authors:** Juneyoung Lee, Louise D. McCullough

**Affiliations:** Department of Neurology, McGovern Medical School, The University of Texas Health Science Center at Houston, Houston, Texas, USA.

## Abstract

Stroke is a leading cause of death and long-term disability. T cells have been extensively studied for their dual role in regulating immunity and inflammation following stroke. In this issue of the *JCI*, Cai, Shi, et al. demonstrated that CD8^+^ regulatory-like T cells (CD8^+^ TRLs) are one of the earliest lymphocyte subtypes to enter the brain after experimental ischemic stroke. Using a mouse model of stroke and comprehensive experimental approaches, the authors found that CD8^+^ TRLs reduced both brain damage and functional deficits in both young and aged mice. These unique early responding regulatory T cells may also play a role in a wide array of other T cell–mediated neurological disorders.

## T cell immunology in stroke

Stroke is a leading cause of disability and death (1). Ischemic stroke is the predominant form of stroke, primarily caused by thrombosis or embolism that leads to a lack of blood flow to the brain. Soon after the onset of ischemia, cells in the brain become damaged and undergo cell death, resulting in long-term behavioral deficits. Clinically, mechanical thrombectomy and recombinant tissue-type plasminogen activator (rtPA; the only FDA-approved drug to treat acute ischemic stroke) are effective therapies for restoring the blood flow and salvaging the penumbra (2, 3); however, additional treatment strategies for stroke are still needed, as many patients are ineligible for current therapies. Importantly, recent studies have emphasized that understanding the immunological and inflammatory mechanisms triggered by stroke is a prerequisite for developing immune-based therapeutics.

Preclinical studies using rodent stroke models have provided us with detailed mechanistic insights into how immune responses contribute to stroke outcome and poststroke recovery. Through characterization of the neuroimmune landscape, it is now well documented that stroke can induce both innate (acute phase) and adaptive (chronic phase) immune responses (4). Among many cell types implicated in stroke-induced inflammation, T cells have received much attention due to their importance in regulating both arms of immunity. In patients, T cells are found in the brain tissue within the first one to three days after stroke (5). Several studies using mouse models have further demonstrated both detrimental and protective roles of T cells after stroke. For instance, γδ T cells have been highlighted due to their detrimental role in neuroinflammation. They can be found in infarcted tissue within days following stroke and contribute to inflammation and neurological deficits. These cells secrete the proinflammatory cytokine IL-17, leading to chemotactic neutrophil infiltration (6, 7). In contrast to γδ T cells, regulatory T (Treg) cells facilitate recovery by reducing inflammation (8). In mice, manipulation of Treg cells using both adoptive cell transfer and depletion models showed Treg-mediated neuroprotection due to enhanced secretion of antiinflammatory cytokines, including IL-10 (8). Very recent studies have revealed that inflammation in peripheral tissues, such as the gut, can regulate T cell–mediated inflammation after stroke (9, 10). Thus, targeting T cells may be a promising therapeutic option for stroke.

## CD8^+^ regulatory-like T cells as protective cells for stroke

T cells are highly immunomodulatory in the poststroke brain. Previous reports have shown that CD8^+^CD122^+^ T cells perform regulatory functions in autoimmune and inflammatory diseases (11–15). In this issue of the *JCI*, Cai, Shi, et al. (16) focused on the role of CD8^+^CD122^+^ T cells in stroke. They identified a subtype of CD8^+^CD122^+^CD49d^lo^ T cells as CD8^+^ regulatory-like T cells (CD8^+^ TRLs) that enhanced neuroprotection after stroke. Using a transient middle cerebral artery occlusion (tMCAO) model in mice, they showed that prior to infiltration of CD4^+^ Treg cells, CD8^+^ TRLs infiltrated the brain as early as one day after stroke and peaked at poststroke day five. These cells remained in the brain for at least two weeks after injury and highly expressed HELIOS and IL-10, which are essential for the regulatory functions of T cells (17). To directly determine the contribution of these CD8^+^ TRLs, Cai, Shi, et al. selectively depleted these cells using an anti-CD122 monoclonal antibody that does not affect other immune cells. CD8^+^ TRL–depleted mice had larger infarcts, acutely and chronically, after ischemia, with worse behavioral outcomes. Importantly, these poststroke characteristics were reversed by CD8^+^ T cell reconstitution using FACS-isolated CD8^+^CD122^+^CD49d^lo^ TRLs adoptively transferred into mice depleted of CD8^+^ TRLs two hours after stroke (16).

**Figure 1:**
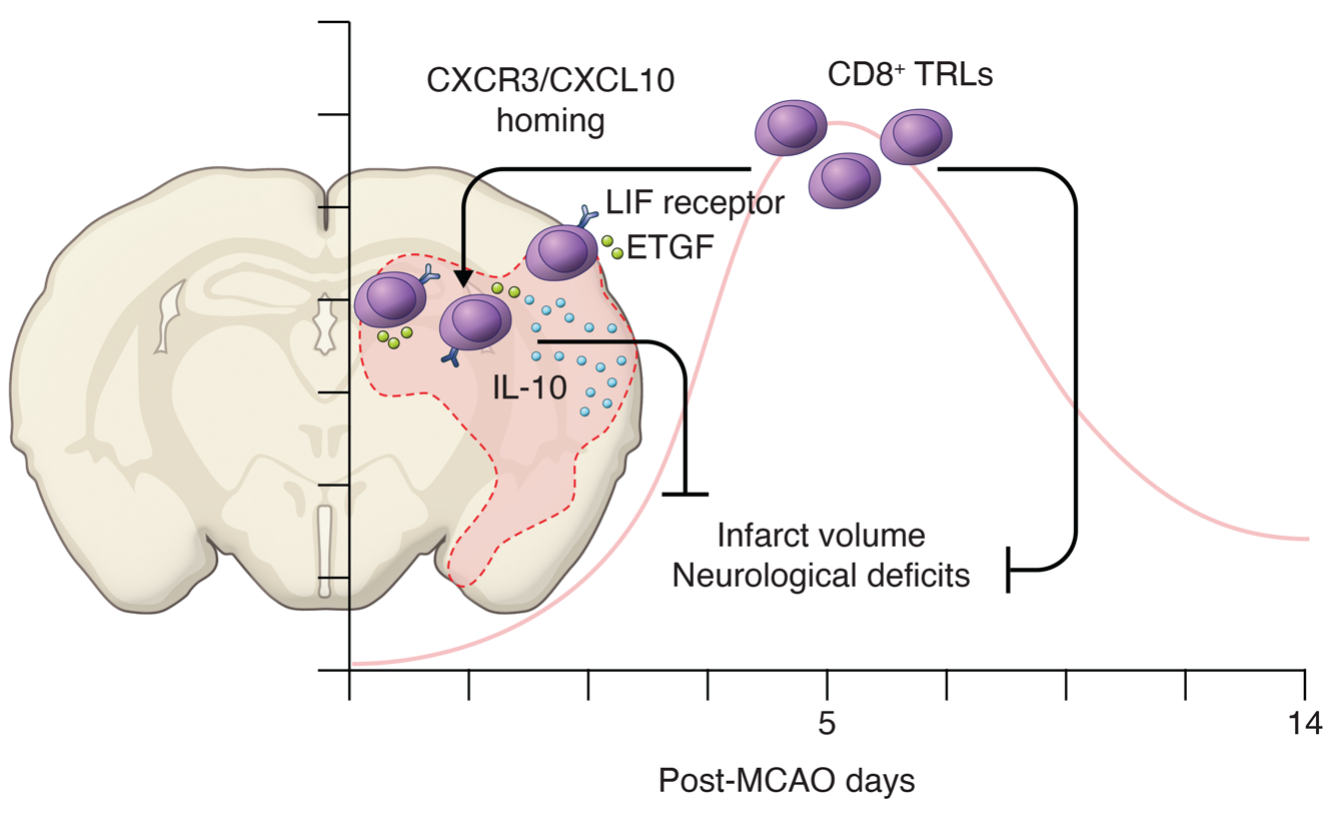
CD8^+^ TRLs confer early protection in the ischemic brain following stroke. Cai, Shi, et al. (16) showed that brain CD8^+^ TRLs are remarkably increased in the early phase of stroke, contributing to the amelioration of brain damage and improving functional recovery in mice. CXCR3 was upregulated in circulating CD8^+^ TRLs and CXCR3-CXCL10 interactions were critical for the infiltration of the ischemic brain tissues by CD8^+^ TRLs. Days after stroke, CD8^+^ TRLs from the ischemic site showed increased LIF receptor, ETGF, and IL-10, which critically regulated the neuroprotective effect. Figure illustrated by Rachel Davidowitz.

Subsequent experiments including RNA sequencing and the use of transgenic mouse models revealed that the interaction between CXCR3 and CXCL10 played an important role in the homing of these CD8^+^ TRLs into the brain after stroke. Once these cells entered the brain, they upregulated leukemia inhibitory factor receptor (LIFR), epidermal growth factor–like transforming growth factor (ETGF; the protein product of *TGFA*), and IL-10 within the ischemic hemisphere (Figure 1). The authors convincingly demonstrate that the neuroprotective effect of CD8^+^ TRLs was mediated by activation of the ETGF and IL-10 pathways through interactions with LIF/LIFR. Of note, LIF signaling is critical for the regulatory function of T cells (18). Indeed, pretreatment with a LIFR inhibitor abolished the protective effects of CD8^+^ TRLs. Using an oxygen-glucose deprivation model, they further showed that CD8^+^ TRL–conditioned media can directly protect primary neurons in an ETGF-dependent manner. Finally, the authors found that adoptive transfer of sorted CD8^+^ TRLs into poststroke mice is beneficial and improved behavioral outcomes in both young male mice and aged mice of both sexes. This beneficial effect was also seen in young male recipient mice adoptively transferred with CD8^+^ TRLs from aged (20-month-old) donor mice, suggesting that the beneficial function of these cells is retained with aging.

## Unanswered questions and conclusions

Certain classes of T cells are detrimental in the early phase of stroke (19, 20). Among the regulatory niche of immune cells, Cai, Shi, et al. (16) have elegantly demonstrated that CD8^+^ TRLs confer neuroprotection, although multiple interesting questions remain: (a) What environmental cues in the periphery or in the injured brain lead to the infiltration of CD8^+^ TRLs in the early phase of stroke? (b) How do these CD8^+^ TRLs crosstalk with other immune cells, including microglia? (c) Are there interactions between CXCR3, LIFR, and ETGF, which appear to be master regulators of the function of CD8^+^ TRLs, in the postischemic brain? (d) Is the change in CD8^+^ TRLs seen in this study specific to stroke-induced inflammation? (e) Does the enhancement of CD8^+^ TRLs alter the systemic immune response or the risk of poststroke infections? Examining whether this population of cells shows similar findings in stroke patients and how these cells change in both the periphery and the brain utilizing samples from stroke patients will be an important next step. This study by Cai, Shi, et al. (16) confirms that T cells are important in the response to brain ischemia, and specific subsets of T cells remain players in the orchestrated immune response to stroke.

## Version 1 08/01/2022

Electronic publication
